# Environmental trade-offs of pig production systems under varied operational efficiencies

**DOI:** 10.1016/j.jclepro.2017.07.191

**Published:** 2017-11-01

**Authors:** G.A. McAuliffe, T. Takahashi, L. Mogensen, J.E. Hermansen, C.L. Sage, D.V. Chapman, M.R.F. Lee

**Affiliations:** a Rothamsted Research, North Wyke, Okehampton, Devon EX20 2SB, UK; b School of Veterinary Sciences, University of Bristol, Langford, Somerset BS40 5DU, UK; c Department of Agroecology, Aarhus University, Blichers Allé 20, DK-8830 Tjele, Denmark; d Department of Geography, University College Cork, Donovan's Road, Cork, Ireland; e School of Biological, Earth and Environmental Sciences, University College Cork, Distillery Field, North Mall, Cork, Ireland

**Keywords:** Pig production, Environmental footprint, Life cycle assessment, Feed composition, Feed conversion ratio

## Abstract

Production of pork, the most consumed meat globally, is estimated to emit 668 m tonnes CO_2_-eq of greenhouse gases each year. Amongst various production systems that comprise the pig industry, grain-based intensive production is widely regarded as the largest polluter of the environment, and thus it is imperative to develop alternative systems that can provide the right balance between sustainability and food security. Using an original dataset from the Republic of Ireland, this paper examines the life-cycle environmental impacts of representative pig farms operating under varying production efficiencies. For the baseline farm with an average production efficiency, global warming potential (GWP), acidification potential (AP) and eutrophication potential (EP) per kg carcass weight departing the slaughterhouse were estimated to be 3.5 kg CO_2_-eq, 43.8 g SO_2_-eq and 32.1 g PO_4_-eq, respectively. For herds with a higher production efficiency, a 9% improvement in feed conversion ratio was met by 6%, 15% and 12% decreases in GWP, EP, AP, respectively. Scenario and sensitivity analyses also revealed that (a) a switch to high-protein diets results in lower GWP and higher AP and EP, and (b) reducing transportation distances by sourcing domestically produced wheat and barley does not lower environmental impacts in any notable manner. To improve cross-study comparability of these findings, results based on an auxiliary functional unit, kg liveweight departing the farm gate, are also reported.

## Highlights

• Environmental footprints of Irish commercial pig production systems were examined.
• Dual-functional unit approach was adopted to enhance cross-study comparability.
• Trade-offs were identified between global warming and acidification/eutrophication.
• ‘Feed mileage’ was found to have little impact on environmental performance.
• Economic and environmental performances were shown to be positively correlated.

## 1 Introduction

Agricultural production is one of the key anthropogenic activities where environmental burdens can potentially be reduced. The farming sector occupies 30% of the Earth's terrestrial surface (Steinfeld, 2006) and 75% of this land use is associated with livestock production (Cassidy et al., 2013). Food systems generate 19–29% of global greenhouse gas (GHG) emissions, of which various forms of primary production contribute 80–86% (Vermeulen et al., 2012). Changing diets and population growth have been associated with 65% of land use change between 1961 and 2011 (Alexander et al., 2015), and demand for livestock products will continue to exceed expected population growth (34%) at least until 2030 because of the ongoing dietary shifts in developing countries (Havlík et al., 2014).

While it is widely accepted that ruminants are the primary drivers of agriculture-related global warming through enteric fermentation, recent evidence suggests that production of monogastric animals also require significant attention, as they too compete for human edible-food for land resources. In particular, pork is the most consumed meat globally (OECD, 2017), and its production is estimated to emit 668 M tonnes CO_2_-eq yr^−1^, or 9% of total livestock emissions (Gerber et al., 2013). Given the continuing globalisation of food and feed markets and the upward pressures on farmland prices, it is imperative to develop pig production systems that provide the right balance between economic, environmental and societal sustainability and food security. To date. studies have demonstrated that improved sow efficiency, through higher numbers of piglets born alive and reduced dry periods, can decrease environmental burdens (Reckmann and Krieter, 2015). Furthermore, higher feed conversion efficiency (FCE) has also been shown to reduce the environmental impact per pig unit, as emissions and losses associated with the feed production stage become smaller (Nguyen et al., 2011). However, published research investigating these effects on the system-wide footprint is rather limited, and thus the environmental benefit of economically improved pig operations is not clearly understood.

Using the life cycle assessment (LCA) framework, which has been applied to a diverse range of pig production systems as reviewed by McAuliffe et al. (2016), the present study investigates the environmental performances of intensive pig production systems in the Republic of Ireland (RoI) under different production efficiencies. Pig production is the third most important agricultural sector in RoI based on gross agricultural output (Teagasc, 2016). Contrary to the country's beef and dairy sectors that have frequently been examined for their environmental impacts (Casey and Holden, 2005, Casey and Holden, 2006), and despite nationwide discussions on the merits of LCA in national GHG evaluations (Schulte et al., 2011), Irish pig production has not been the subject of a systems study to date. As of June 2015, there were 1.54 million pigs in RoI and, with an annual net production of just over 276,000 tonnes, the national self-sufficiency rate was 195%; nearly half of total production was exported. Although the Irish pig industry is relatively small compared to some of the EU ‘powerhouses’, it has the highest exporting percentage to non-EU countries within the union (Forde, 2016) and thus is strongly linked to the international market. For this reason, the majority of findings from the present study are likely to be also applicable to pork supply chains elsewhere.

Similarly to continental Europe, most pig production in RoI occurs on large-scale integrated units, where piglets are born, weaned and fattened on the same farm. On these farms, feed is typically purchased from specialised production mills, but with the recent volatility of international cereal prices, a small number of Irish pig farmers have constructed their own on-farm mills to minimise costs and maximise nutritional control over their feed formulations. In addition to the baseline analysis whereby feed is assumed to be mass-produced, the present study investigates the effect of this ‘local feed’ movement on the environment footprint. While a range of LCA studies have considered differences in feed composition (Garcia-Launay et al., 2014, Ogino et al., 2012, Stone et al., 2012), no identified studies have considered the location and the ownership of feed mills.

In RoI, 7.4% of the total agricultural land is used for arable crop production and the country is close to self-sufficiency (encompassing human, animal and industrial uses) for major cereals (DAFM, 2009). However, many feed mills source a significant portion of cereal ingredients from overseas, especially when the international market is in a favourable condition (in regard to cereal prices and exchange rate). Replacing these cereals with domestically grown barley (*Hordeum vulgare*) and wheat (*Triticum* spp.) could potentially contribute to lower total transport distances, more efficient use of manure (nutrient balancing) and, perhaps to a lesser extent, long-term food security. The present study tests this hypothesis by investigating whether the reduced transportation, when coupled with domestic conditions for crop production (and the associated emissions), would alter the overall LCA results. Finally, four sets of sensitivity analyses are conducted to evaluate the consequences of different allocation methods as well as alternative assumptions regarding land use change (LUC), utilisation of pig manure by crop farmers, and on-farm energy usage.

## 2 Materials and methods

In this study, LCA was applied to the Irish pig industry under both a typical industry setting (baseline analysis) and altered production systems (scenario analyses).

### 2.1 Goal, scope and functional unit

**Fig. 1:**
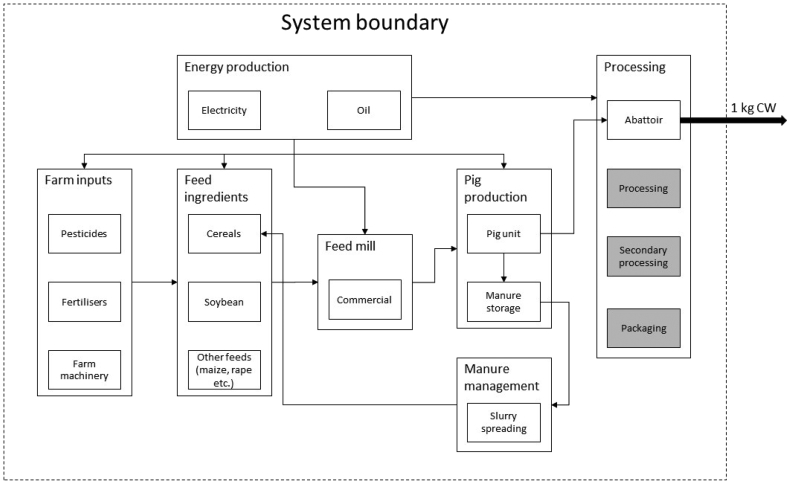
Stylised schematic of the baseline study boundary. Grey processes are excluded from analysis.

The primary goal of this study was to compare environmental performances of intensive pig production units operating at different efficiencies and evaluate the effectiveness of alternative strategies to improve sustainability of the industry. The system boundary for the baseline analysis was set as being from the ‘cradle’, or the production of input materials, to the end of the slaughtering process (Fig. 1). The environmental performance of each system was evaluated with the functional unit of 1 kg carcass weight (CW), of finishers and cull sows combined, as measured at the time when the intermediate product (dressed carcass) exits from the slaughterhouse. This functional unit was adopted to represent a wide range of pigmeat both (a) directly sold to retailers, as well as (b) initially distributed for secondary processing. Consequently, secondary processing and supply chain distribution beyond the abattoir were excluded from the model. Previous research reviewing global pig LCA studies has noted, however, that cross-system comparison of environmental performances is extremely challenging when scopes and functional units are not shared between different analyses (McAuliffe et al., 2016). Motivated by this criticism, outputs based on the auxiliary functional unit of 1 kg liveweight (LW), as measured at the time when the intermediate product (live animal) exits from the farm gate, is also reported in this study. For the conversion of LW to CW, a kill-out rate of 76% (Teagasc, 2014) was assumed.

### 2.2 Life cycle inventory analysis (LCI)

#### 2.2.1 Feed production

Feed composition data for the baseline analysis were obtained from a large-scale commercial mill in RoI. These data were representative of commercially available feed rations used in Irish pig industry during February 2015. Diet formulations were distinguished between dry sows, lactating sows, weaners and finishers, and replacement gilts that have reached the finishing weight but are yet to be served were assumed to consume the same amount of feed as dry sows. The major ingredients for these feed rations included barley, maize (*Zea mays*), soybean (*Glycine max*) products and wheat (Table 1). All rations were formulated using the principle of least-cost rationing and balanced for macro and micro nutrients through the addition of supplements (mineral premix and synthetic amino acids: SAA) to meet animal requirements. Environmental implications of using premix supplements were considered to be the same as the production of calcium carbonate (Mosnier et al., 2011). Environmental burdens of SAA, of which mass accounted for <1% of total mass of ration, were excluded from the current LCA model due to unavailability of commercial sensitive data pertaining to the exact SAA composition within the recipe. A similar approach has been employed by Dourmad et al. (2014) and Nguyen et al. (2010) and, while SAA have a large environmental footprint when evaluated on a per kg basis (Garcia-Launay et al., 2014, Mosnier et al., 2011), those arising from their production process at a system level are generally small due to minimal quantities mixed into the feed (Strid Eriksson et al., 2005). No medicines or growth-promoting agents were included in the compound feed rations. The nutritional composition of the feed ingredients presented in Table 2 was compiled based on data from FAO (2015). Background data for crop production together with associated yields and environmental burdens were sourced from the Agri-footprint database (Blonk Consultants, 2015), in which impacts of pesticide application events were considered, while those of upstream production were not. Based on data provided by the mill manager, it was assumed that 11 kWh of national grid electricity were used to produce 1000 kg of the mixed feed. As this feed was wet-mix, heat was not required for compression.

**Table 1:** Feed composition for pig diets.

Ingredient (kg/1000 kg)	Origin^a^	Baseline analysis				Scenario analysis (on-farm feed mill)			
		Dry sow	Lact. sow	Weaner	Finisher	Dry sow	Lact. sow	Weaner	Finisher
Barley	IE	210	240	180	240				
	UK					350	320	350	362
Beet pulp	FR					80	20	20	25
Maize	FR	220	220	230	255	60	80	120	150
Premix	UK	25	25	25	20	28	40	35	28
Rapeseed meal	DE	70	30	40	85				
Soybean hulls	AR	50			15	50			
Soybean meal	AR	90	200	220	120	143	195	242	165
Soybean oil	AR	25	35	35	25	5	25	26	
Wheat	IE	44	80	108	58				
	FR	200	50		95				
	UK	66	120	162	87				
	DK					284	320	207	270

a IE: Ireland, UK: United Kingdom, FR: France, DE: Germany, DK: Denmark, AR: Argentina.

**Table 2:** Nutritional composition of individual feed ingredients (FAO, 2015), crop yields in primary production (Blonk Consultants, 2015), and transportation distances.

Ingredient	Origin^a^	DM^b^ (%)	CP (%)	P (g kg DM^−1^)	K (g kg DM^−1^)	Yield (kg DM ha-1)	Sea distance (km)	Road distance (km)^c^
Barley	IE	87.1	11.8	3.9	5.7	7050		93
	UK	87.1	11.8	3.9	5.7	5710	1413	88
Beet pulp	FR^d^	89.2	9.3	1	4.5	8920	832	229
Maize	FR	86.3	9.4	3	3.9	9030	832	145
Rapeseed meal	DE	91	34.1	11.5	12.5	3750	1428	319
Soybean hulls	AR	89.1	13.2	1.6	13.7	2440	11647	379
Soybean meal	AR	87.9	51.8	6.9	23.7	2440	11647	379
Wheat	IE	87	12.6	3.6	4.6	8570		22
	FR	87	12.6	3.6	4.6	6980	832	408
	UK	87	12.6	3.6	4.6	7480	454	374
	DK^d^	87	12.6	3.6	4.6	7160	2134	336

a IE: Ireland, UK: United Kingdom, FR: France, DE: Germany, DK: Denmark, AR: Argentina.

b DM: dry matter; CP: crude protein; P: phosphorus; K: potassium.

c Based on the distances between the largest arable region for the crop in each country (e.g. Cordoba for Argentinian soybean).

d These crop-origin combinations are used by the on-farm feed mill only.

Information on origins and transportation of crop ingredients was provided by the mill and an importation company. As at February 2015, soybean products were imported from Argentina and shipped from Rosario Harbour. French maize, wheat and beet (*Beta vulgaris*) pulp were transported from Boulogne. Barley and wheat from the UK were delivered from Liverpool, while premix supplements were transported by road from Belfast. All sea-based cargo was delivered to Ringaskiddy harbour in Cork, RoI, and the nautical distances were calculated using Portworld (2016). From Ringaskiddy, these ingredients were transported using trucks, and the road-based distance for this segment was calculated using a geographical information system (Table 2).

The environmental burdens arising from crops with multiple outputs were allocated by means of economic allocation. While splitting the responsibility of downstream emissions and losses into multiple upstream production processes could potentially disrupt mass and energy balances (Weidema and Schmidt, 2010), system expansion to cover the entire value chain of upstream products such as soybean oil and rapeseed (*Brassica napus*) meal was considered to be impractical given the scope of the present study (Ardente and Cellura, 2012). Following the recommendation by preceding studies that assignment of environmental burdens between crop co-products is best carried out by way of economic allocation (Williams et al., 2006), this method was adopted for background crop processes of the baseline analysis. Economic values of co-products were adopted from the Agri-Footprint database (Blonk Consultants, 2015), of which primary data originate from Vellinga et al. (2013).

#### 2.2.2 Pig production

Herd performance data were based on national statistics compiled by Teagasc (2014). These data covered 84,000 sows or 56% of the national breeding population. While farm size in the original record ranged from less than 100 sows to over 2500 sows, the present study was carried out for the average herd size of 752 sows. Three sets of productivity data were used in this study (Teagasc, 2014): those representing farms with an average herd performance (AVG), the top 25% farms (T25) and the top 10% farms (T10), as measured by the number of pigs produced per sow and FCE of growing pigs. Consequently, three representative farms were set up for the baseline analysis (Table 3).

**Table 3:** Performance data for three levels of productivity; average herd performance (AVG), the top 25% (T25) and the top 10% (T10).

Parameter	Unit	AVG	T25	T10
*Breeding herd*				
Sows	n	752	752	752
Replacement rate	%	50	52	48
Gilts	n	411	415	385
Sow mortality	%	5.1	3.7	3.7
Total litters per sow	n	4.3	4.4	4.7
Piglets per litter	n	13	13	13
Empty days	d	14	9.0	7.0
Sow liveweight	kg	250	250	250
Sow carcass yield	%	69	69	69
Feed consumed as dry sow	kg	1930	1980	2075
Feed consumed as lactating sow	kg	422	451	480
Feed consumed as gilt	kg	345	357	375
*Growing pigs*				
Weaning weight	kg	7.0	7.0	7.0
Weaner mortality	%	2.6	1.8	1.2
Feed consumed per weaner	kg	55	55	49
Finisher culling weight	kg	106	108	108
Finisher mortality	%	2.4	2.0	1.5
Finisher carcass yield	%	76	77	76
Feed consumed per finisher	kg	195	175	180
Total growing period	d	176	172	175

Herd dynamics, including the schedule of replacement, was mathematically estimated for each of the three representative farms under the assumption that they are operating at steady state. Adult males were excluded from the inventory because of the disproportionally large number of sperm doses produced by a single boar under artificial insemination systems (Knox, 2016). The derived information shows that animals on the T10 herds tend to stay on farm for a longer period of time than the T25 herds, but meanwhile consume less than the T25 farms (Table 3). The number of piglets born alive per sow was highest for T10, and this led to the higher sow feed intake, particularly at the farrowing stage (Teagasc, 2014). The T10 herds also had the lowest mortality rates across all stages of production. Carcass yields between the three categories were similar, suggesting that the difference in production efficiency is mostly attributable to better management of nutrition and health, rather than the difference in the target market. Based on local data provided by McCutcheon (2012), energy usage on farm was assumed to be 28 kWh per head (including both sows and finishers), of which 53% was consumed in the form of metered electricity and 47% in the form of processed light fuel oil used predominately for underfloor heating and ambient temperature regulation.

Pig manure in RoI is typically utilised as an organic fertiliser. On the majority of pig farms, animals are housed on slatted floors, where manure drains, assisted with water hosing, into an underground storage tank. Manure is usually stored in temporary tanks for less than one month, and then pumped out to an outside storage tank where it remains until receiving farmlands are ready for nitrogen (N) application. The pig units are typically large-scale indoor enterprises, and most pig farmers do not own enough land for arable production to spread the entire manure-output on (Nolan et al., 2012). Consequently, the manure is often transported to nearby arable farms for utilisation. In this study, it was assumed that manure was transported 10 km to receiving farmland. Diesel energy required for spreading manure was assumed to be 21 MJ per 1000 kg (Nguyen et al., 2010, Reckmann et al., 2013), mostly attributable to the use of a tractor and manure spreader. Both the positive and negative effects of pig manure were considered in the baseline analysis, the former as a cause to reduce the demand for manufactured fertiliser and the latter as a source of ammonia (NH_3_), methane (CH_4_), nitrate (NO_3_^−^), nitrous oxide (N_2_O) and phosphate (PO_4_^3−^) losses.

#### 2.2.3 Slaughterhouse process

Most LCA studies that include the slaughterhouse within the system boundary demonstrate that, in comparison to primary production, the environmental impacts arising from this process are minor (Nguyen et al., 2011, Reckmann et al., 2013). Since primary data from Irish slaughterhouses were unavailable, data for the slaughtering process were taken from Reckmann et al. (2013), as their production environment in Germany was deemed most similar to the Irish situation. These authors report energy usage and emissions associated with the abattoir, while assuming that waste products and by-products are disposed of as biodegradable materials. Detailed inventory data prepared for the baseline analysis can be found in Supplementary Table S1. Water use was not included due to the finding by Reckmann et al. (2013) that it had minimal impacts on GWP, AP and EP. The carcass yield (kill-out %) for each representative farm, obtained from Teagasc (2014), is listed in Table 3.

#### 2.2.4 Emissions and losses

Emission factors used in this study are provided in Table 4. The parameters for CH_4_ emissions were taken from the Irish National Inventory Report (Duffy et al., 2017), while N_2_O emissions were calculated using IPCC (2006) guidelines. NO_X_ and NH_3_ emissions were calculated according to the methodology reported in Nguyen et al. (2011). Nutrient contents in manure were estimated using the nutrient balance, where the N, P and K contents in body tissues were subtracted from those in feed (Poulsen et al., 2001). Once applied to farmland under typical Irish conditions, 50% of manure N and 100% of manure P and K were assumed to become available for plant uptake (Government of Ireland, 2010). For P, 3% of this value is assumed to become lost through leaching (Nguyen et al., 2011).

**Table 4:** Emission factors adopted in the current study.

Pollutant	Emission factor	Reference
*CH*_*4*_		
Enteric fermentation (kg CH_4_ head^−1^ year^−1^)		Duffy et al. (2017)
Gilts (in pig)	2.9	
Gilts (not served)	2.2	
Sows (in pig)	3.7	
Other sows	3.8	
Growing pigs > 20 kg	1.1	
Growing pigs < 20 kg	0.2	
Manure management (kg CH_4_ head^−1^ year^−1^)		
Gilts (in pig)	8.0	
Gilts (not served)	5.0	
Sows (in pig)	8.0	
Other sows	18.8	
Growing pigs > 20 kg	5.1	
Growing pigs < 20 kg	3.4	
*Direct N*_*2*_*O-N*		
Manure management		
In-house storage	0.002 × kg manure N ex-animal	IPCC (2006)
Outside storage with natural crust	0.005 × kg manure N ex-housing	
Field application	0.01 × kg manure N ex-storage	
Fertiliser application	0.01 × kg fertiliser N	
*NO*_*x*_*-N*		
Manure management		
In-house storage	0.002 × kg manure N ex-animal	Dämmgen and Hutchings (2008)
Outside storage	0.005 × kg manure N ex-housing	
Field application	0.001 × kg manure N ex-storage	Nemecek and Kägi (2007)
Fertiliser application	0.007 × kg fertiliser N	EEA (2007)
*NH*_*3*_*-N*		
Manure management		
In-house storage	0.13 × kg manure N ex-animal	Nguyen et al. (2010)
Outside storage	0.02 × kg manure N ex-housing	
Field application	0.07 × kg manure N ex-storage	Andersen et al. (2001)
After field application	0.117 × kg manure N ex-storage	Hansen et al. (2008)
Fertiliser application	0.065 × kg fertiliser N	Nguyen et al. (2010)
*NO*_*3*_*-N leaching potential*	kg N ex-animal - kg N total N loss - kg fertiliser N substitution	Nutrient balance
*PO*_*4*_*-P leaching potential*	kg P ex-animal - kg fertiliser substitution	
*Indirect N*_*2*_*O-N*	0.01 × kg (NH_3_-N + NO_x_-N) loss + 0.0075 × kg NO_3_-N	IPCC (2006)

The reduction in GHG emissions due to avoided production of manufactured fertiliser was estimated to be 6.6 kg CO_2_-eq/kg fertiliser N (Weidema et al., 2014), 2.7 kg CO_2_-eq/kg fertiliser P and 0.8 kg CO_2_-eq/kg fertiliser K (Nielsen et al., 2007). Energy savings associated with reduced on-farm activities were assumed to be 0.4 MJ diesel/1000 kg fertiliser N (Nguyen et al., 2011; based on Dalgaard et al., 2001). The associated reduction in emissions from soil was also accounted for (Table 4). Reduced emissions from P and K application were not included in the model due to their small quantities, which were assumed to be spread together with N fertiliser. The complete LCI data for 1000 kg LW at the farm gate are given in Table 5.

**Table 5:** LCI inputs and outputs for 1000 kg LW at the farm gate.

Item	Unit	Baseline analysis			Scenario analysis (on-farm feed mill)		
		AVG	T25	T10	AVG	T25	T10
*Feed use*	kg						
Dry sow		339	326	308	339	326	308
Lactating sow		74	70	71	74	70	71
Gilt		61	59	56	61	59	56
Weaner		517	514	453	517	514	453
Finisher		1790	1590	1640	1790	1590	1640
Total		2781	2559	2528	2781	2559	2528
*Transport of feed (from mill)*							
By truck	Tkm	313	288	285	0	0	0
*Energy use*							
Electricity	kWh	137	135	136	137	135	136
Heat (oil)	kWh	121	120	120	121	120	120
*On-farm emissions*							
Methane	kg						
Enteric fermentation		5.0	5.0	5.0	5.0	5.0	5.0
Manure management		63	61	62	63	61	62
Nitrous oxide	g	301	258	249	446	390	376
Ammonia	kg	5.4	4.6	4.5	8.0	7.0	6.8
Nitrogen oxides	g	631	539	520	933	817	785
*Manure utilisation*							
Transport	Tkm	72	62	60	107	94	90
Spreading	MJ	152	130	125	224	196	189
Nitrous oxide	g	669	572	552	989	866	832
Ammonia	kg	5.8	4.9	4.8	8.6	7.5	7.2
Nitrogen oxides	g	84	72	69	124	108	104
Nitrate	kg	45	39	37	70	59	56
Phosphate	g	222	164	156	385	314	304
*Avoided fertiliser production*	kg						
from manure nitrogen		39	33	32	57	50	48
from manure phosphorus		11	8.0	8.0	19	15	17
from manure potassium		26	24	23	35	32	32
*Avoided fertiliser application*							
Spreading	MJ	15	13	13	23	20	19
Nitrous oxide	g	161	137	132	237	208	200
Ammonia	kg	0.8	0.7	0.7	1.2	1.0	1.0
Nitrogen oxides	g	235	201	194	347	304	292

### 2.3 Impact assessment and interpretation

SimaPro 8.1 (PRé Consultants, 2016) was used to model the studied systems. The three impact categories previously identified to be important for pig LCA studies (McAuliffe et al., 2016), namely global warming potential (GWP), acidification potential (AP) and eutrophication potential (EP) were estimated for the three representative farms with varying levels of productivity using the CML (2013) baseline impact assessment method. The outputs for the baseline analysis were expressed, respectively, in units of kg CO_2_-eq kg CW^−1^, g SO_2_-eq kg CW^−1^ and g PO_4_-eq kg CW^−1^. Of the various sources of uncertainties surrounding LCA outputs, the effect of those inherent in livestock performance and farm management was assessed through (a) the comparison of the three representative farms as discussed in Section 2.2, and (b) a range of scenario and sensitivity analyses as outlined below. Furthermore, the effect of uncertainties related to on-farm emissions was evaluated by means of Monte Carlo analysis and the resultant outputs were compared pairwise between the three representative farms. For the latter procedure, parameters were randomly drawn over 1000 iterations from the distributions summarised in Supplementary Table S2.

#### 2.3.1 Scenario analyses

For the first scenario analysis to examine the environmental implications of on-farm feed milling (see Section 1), data were collected from a small-scale farm-operated mill in the south of RoI. The data inventory presented on the right-hand side of Table 1 replaced the baseline inventory for this analysis. Based on information provided by the mill manager, it was assumed that 30 kWh of electricity was used to process 1000 kg of feed, the level far above what was assumed for the large-scale specialist mill (11 kWh) in the baseline analysis. Since the mill is located adjacent to the piggery, the on-road transportation process linking the feed mill to the representative farms was eliminated from the model (Table 5). For the second scenario analysis to examine the consequences of reduced transport distances, all imported cereals in the baseline inventory were replaced by domestically produced counterparts of the same quantity. To be consistent with the baseline analysis, data related to domestic crop production were also sourced from Agri-footprint (Blonk Consultants, 2015).

#### 2.3.2 Sensitivity analyses

The economic allocation method was used in the baseline analysis to separate environmental burdens associated with crops with more than a single material flow. A sensitivity analysis was conducted, here using mass-allocation, in order to test the robustness of the baseline results. This analysis was performed on all crops that had multiple outputs; for example, meal and oil from soybean and rapeseed.

Due to the relatively small scale of the Irish pig industry, the baseline analysis of the present study assumed that changes in feeding strategy on Irish farms would not cause LUC elsewhere in the world. Recent research has shown, however, that the inclusion of LUC in the assessment of soybean production systems can increase the resultant GHG emissions by as much as nine-fold when the entire crop-growing area is assumed, somewhat unrealistically, to have been forest previously (Maciel et al., 2016). Under a more reasonable assumption, a UK study by Audsley et al. (2009) posited that, when LUC is included in the model, up to 40% of the country's food-sector emissions would originate outside the country. Given the significance of such a potential impact, a sensitivity analysis to examine the potential effect of LUC was conducted using information compiled by Blonk Consultants (2015) in conjunction with PAS2050-1 (BSI, 2012). Emissions arising from LUC were estimated for rapeseed (Germany), soybean (Argentina) and wheat (RoI, Denmark and the UK). For production of barley (RoI and the UK), maize (France) and sugar beet (France), land transformation was deemed unnecessary (Blonk Consultants, 2015).

In addition, several on-farm assumptions were deemed to require sensitivity analyses. First, the inclusion of the fertiliser offsetting effect in the baseline analysis (where manure N, P and K replace inorganic nutrients) implicitly assumes that pig manure is perfectly utilised by receiving farmers. Although pig manure is a useful by-product, it is difficult in reality to match demand and supply without wastage. Therefore, a sensitivity check was conducted to examine the effect of this offsetting on the overall results by assuming the other extreme case, whereby manure is applied to arable land in addition to manufactured fertilisers (i.e. in excess of crop nutrient requirements), resulting in no reduction in fertiliser production. Additionally, while the on-farm energy usage in this study was assumed to be 28 kWh per head, preceding studies show that this value ranges widely across pig farms in RoI. Thus, using the upper limit (45 kWh per head) and lower limit (18 kWh per head) reported by McCutcheon (2012), two additional versions of models with high and low energy usage (retaining the electricity–fuel oil ratio of 53:47) were generated to examine the effects of this value on the overall environmental footprint.

## 3 Results and discussion

The environmental impact per kg CW obtained from the baseline analysis is displayed in Table 6. A detailed breakdown of contributions from all system processes is provided as the supplementary material (Tables S3–S5).

**Table 6:** LCIA results for the baseline analysis expressed per 1 kg carcass weight (CW) for three different levels of productivity: average herd performance (AVG), the top 25% (T25) and the top 10% (T10).

	AVG				T25				T10			
	Feed	Farm	Slaughter	Total	Feed	Farm	Slaughter	Total	Feed	Farm	Slaughter	Total
GWP (kg CO2-eq kg CW^−1^)	2.03 (58%)	1.17 (33%)	0.31 (9%)	3.51	1.86 (56%)	1.14 (35%)	0.30 (9%)	3.30	1.85 (56%)	1.14 (35%)	0.31 (9%)	3.30
AP (g SO2-eq kg CW^−1^)	19.5 (45%)	23.2 (53%)	1.1 (2%)	43.8	17.8 (46%)	20.0 (51%)	1.1 (3%)	38.9	17.7 (46%)	19.3 (51%)	1.1 (3%)	38.1
EP (g PO4-eq kg CW^−1^)	16.2 (50%)	11.8 (37%)	4.1 (13%)	32.1	14.8 (51%)	10.2 (35%)	4.0 (14%)	29.0	14.7 (51%)	9.8 (34%)	4.1 (14%)	28.6

### 3.1 Global warming potential

GWP of the average (AVG) farm was estimated to be 3.5 kg CO_2_-eq/kg CW, with the 95% confidence interval (accounting for uncertainties surrounding on-farm emissions) ranging between 3.3 and 3.8 kg CO_2_-eq/kg CW. Based on the point estimate, the largest GWP hotspot was emissions arising from feed production, accounting for 58% of the total impact (Table 6) at a level comparable to other European studies (MacLeod et al., 2013, Reckmann et al., 2013). Of feed-related impacts, the finisher diet accounted for 65%. Maize had higher emissions than other crops driven primarily by its mass input, wet-mill processing into maize bran and, to a lesser extent, more intensive fertiliser usage when compared to wheat and barley (Blonk Consultants, 2015). Road and sea transport together accounted for 8% of total feed-related emissions. Transportation from Argentina by cargo ship generated 19% of the GWP attributable to soybean products, the only group of feed ingredients originating outside Europe. All other crop ingredients had considerably lower sea transportation impacts (<2%).

On the farm, CH_4_ emissions from manure management and enteric fermentation respectively generated 23% and 5% of total GWP, closely following the results reported by MacLeod et al. (2013). N_2_O emissions arising from manure storage produced 3% of total GWP, while N_2_O emissions from manure application produced 7%. The usage of national grid electricity accounted for 4% of total emissions, while light fuel oil burned in a non-condensing boiler was shown to have a relatively small effect (1%). Of emissions displaced in the arable sector, the reduction of N production resulted in a 9% saving of total emissions; on the other hand, the effect of replacing P and K fertiliser production was less profound (1%). Slaughtering accounted for 9% of total GWP kg CW^−1^, of which electricity was responsible for 79%. This result is similar to the finding by Reckmann et al. (2013), who reported that 7% of total GWP was generated at the slaughterhouse. Contributions from other processes, including farm traction and transport of feed from mill to farm, were all comparatively minimal (Supplementary Table S3).

### 3.2 Acidification potential

AP for the average (AVG) farm was estimated to be 43.8 (41.2–46.5) g SO2-eq/kg CW. NH_3_ emissions from manure storage (indoor and outdoor combined) and application to crop fields respectively accounted for 26% and 28% of the total AP, making NH_3_ losses the largest contributor to this impact category. Avoided NH_3_ emissions from replaced inorganic fertiliser resulted in a 4% decrease from the level of AP that would otherwise have been produced, again insufficient to offset the large emissions arising from manure application. Environmental burdens resulting from NO_X_ were negligible (<1%). Feed production accounted for 45% of the total AP, of which finisher feed represented 66%. These figures are comparable with Nguyen et al. (2011) where feed generated 36% of AP, while Reckmann et al. (2013) reported a slightly lower 23% contribution from feed. In the current study, maize (27%) and barley (26%) were the highest feed-related hotspots. Sea-based transportation accounted for 1.4% of total AP. The slaughterhouse generated 3% of the total AP, of which SO_2_ emissions from combustion during electricity production accounted for 83%.

### 3.3 Eutrophication potential

EP for the average (AVG) farm was estimated to be 32.1 (29.5–35.5) kg PO_4_-eq/kg CW. Feed production was the highest contributor to EP, accounting for 51% of the total value. Similarly to AP, barley and maize were the primary sources, producing 28% and 22% of feed-related burdens, respectively. Losses of eutrophying substances such as NH_3_, NO_3_^−^and PO_4_^3−^ were the primary sources of EP from crop production. NH_3_ emissions from farm management and manure spreading generated 17% of the total EP, while losses of NO_3_^−^ from organic fertiliser application amounted to 19%. Environmental burdens associated with PO_4_^3−^ from manure application on the receiving arable farms were low (1%). The slaughterhouse had a higher impact on EP than GWP and AP, totalling to 13%. The majority (85%) of these burdens stemmed from higher biochemical oxygen demand (BOD), chemical oxygen demand (COD), and increased losses of N and P to water.

### 3.4 Effect of herd performance

As discussed in Section 2.2.2, the three representative farms with different levels of productivity (AVG, T25 and T10) were differentiated by feed intake, mortality, growth rates and, to a lesser extent, carcass yields. Table 6 indicates that improvements in production efficiency generally lead to smaller environmental footprints. Between the average (AVG) farm and the T10 farm, a 9% improvement in feed conversion ratio (from 2.49 to 2.27 kg/kg, as calculated from Table 3) is met by 6%, 12% and 15% decreases in GWP, EP and AP (*p* = 0.06, *p* = 0.03 and *p* < 0.01 based on Monte Carlo pairwise comparisons), respectively. It should be noted, however, that the present method used a fixed emission factor per head for CH_4_ from manure production, which was not adjusted for reduced feed use per kg meat production. These percentages should therefore be seen as the lower limits, rather than the expected values, for the effect of improved farm productivity. Differences in GWP, EP and AP between the average (AVG) and the T25 farms were also found to be systematic (*p* = 0.07, *p* = 0.04 and *p* < 0.01, respectively).

On the other hand, the differences in environmental performances between the two improved herds were not as clear-cut (all *p* > 0.10). The T25 herd finished pigs in less time than T10 while the T10 herd consumed less feed in total (Table 2), leading both their CH_4_ emissions and the overall GWP to be comparable against one another. However, the T25 herd generated more N and caused larger losses of NH_3_ and NO_3_^−^ due to higher feed intake, and as a result larger AP and EP were predicted compared to T10. Thus, environmentally speaking, neither of the improved herds were strictly preferable over the other herd. Economically speaking, lower costs associated with less feed consumption, together with increased throughput of liveweight generally lead T10 to have higher profit margins, followed closely by the T25 herds (Teagasc, 2014). Based on the observation that the farms with higher levels of productivity (T25 and T10) generated lower environmental footprints than the average (AVG) herd, it is plausible to conclude that improvements in animal performance metrics are more likely to be positively correlated with environmental sustainability. This finding is in agreement with Nguyen et al. (2011).

### 3.5 Scenario and sensitivity analyses

**Fig. 2:**
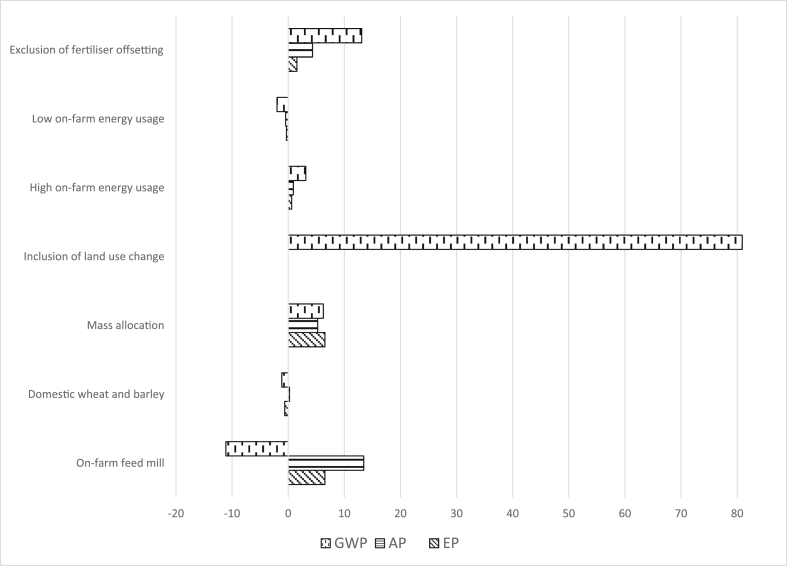
Effect of different analyses on baseline results, presented as percentage change.

Fig. 2 summarises main findings from the scenario and sensitivity analyses for the average (AVG) farm. Detailed results for all three herds (with different production efficiencies) are provided as Supplementary Table S6. All values are reported as percentage change from the baseline results.

#### 3.5.1 Scenario analyses

Some notable differences were observed as a result of replacing feed from the large-scale commercial mill with feed from the small-scale on-farm mill; GWP reduced by 13%, AP increased by 14% and EP increased by 6–7%. These differences between the commercial and on-farm feed mills were largely driven by the ingredients, rather than the milling method. For example, the lower GWP associated with the on-farm mill primarily resulted from lower maize bran usage (associated with 0.82–0.84 kg CO_2_-eq per kg on a dry matter basis) compensated for by larger quantities of cereals (0.34–0.36 kg CO_2_-eq for barley and 0.26–0.33 kg CO_2_-eq for wheat, both on a dry matter basis), a combination that tends to generate a lower carbon footprint due to reduced energy requirements for wet milling of maize (Blonk Consultants, 2015). Although more electricity was used per 1000 kg feed produced at the on-farm mill, this had little impact on the overall GWP (<1%). Increases in AP and EP are explained by the larger quantities of soybean meal included in the diets, which has the highest CP content (51.8%) of all the ingredients. This resulted in larger quantities of N in manure, increasing potential losses of NH_3_ and NO_3_^−^ (Supplementary Tables S7–S9). For example, NH_3_ emissions (measured in g SO_2_-eq) and NO_3_^−^ losses (measured in g PO_4_-eq) were both 33% higher using the on-farm mill diets. These findings alone warrant further research on economic-environmental trade-offs surrounding feeding strategies, as ‘least-cost’ ration formulations are solely driven by the market price of commodities and do not reflect differences in upstream processing requirements or indeed environmental costs attributable to different rations. More immediately, these conflicting results demonstrate the complex nature of interpreting LCA studies and disseminating results to key stakeholders (Guinée et al., 2011). On one hand, advising farmers to include more wheat and barley and less maize (particularly processed maize such as bran) seems to be a logical assessment as the present result suggests subsequent GWP decreases. However, as burdens generated from high protein crops such as soybean products produce higher levels of N in manure resulting in higher AP and EP (Garcia-Launay et al., 2014, Mosnier et al., 2011, Ogino et al., 2012), it is not immediately clear which option is environmentally more desirable when their FCE are comparable. This trade-off needs to be analysed in a local context, taking factors such as the current level of water quality into consideration, before recommendations are communicated with pig producers in the region. In any case, the composition of the ration is a direct consequence of the nutrient demands of the pig and the availability and price of feed ingredients in a particular region, and therefore any potential amendments in formulation are likely to be limited.

Despite lower distances travelled and higher yields achieved under Irish conditions, replacing imported wheat and barley with domestically-grown cereals had minimal effects (1%) across all impact categories. The slight increases observed to GWP and AP are mainly attributable to increased emissions, which was triggered by Irish farmers' general preference towards organic fertilisers (not limited to pig manure) compared to French and UK farmers (Blonk Consultants, 2015). Marginally lower EP values occurred as less NO_3_^−^ was leached on Irish crop farms, due to a higher retention rate of crop residues and less usage of inorganic fertilisers. The present finding that the replacement of imported cereals with domestic crops does not considerably alter the LCA results supports the view by Dalgaard et al. (2007), who argued that the ‘food miles’ concept (Paxton, 1994) was inaccurate and misleading in an environmental context.

#### 3.5.2 Sensitivity analyses

Replacing the economic allocation method with the mass allocation method for all feed crops resulted in GWP and EP increasing by 6%, and AP increasing by 5%. These changes are due to the relatively low economic values (per a given mass) associated to the crop co-products used for pig feed. However, the new output values were largely proportional to the original values and did not affect the relative ranking amongst the three representative farms.

The inclusion of LUC for all crops increased GWP by 78%–81% from the baseline results. These changes were predominantly driven by land transformation from forest to arable land, including CO_2_ emissions of 13,902 kg ha^−1^ for Argentinian soybean production. In the purely local context, because of the relative scale of the Argentinean soybean sector compared to the Irish pig sector, any increase in soybean demand in RoI will more likely be met by destination switch or perhaps altered crop choice rather than development of new arable land. On the global scale, however, the above finding supports the argument by Meul et al. (2012) that, in order to reduce carbon footprints, pig feed producers around the world should minimise LUC sensitive crop ingredients, such as soybean, by adopting low CP diets.

The baseline result showed that reduced production of N fertiliser decreased GWP by approximately 9%. When fertiliser offsetting was excluded from the model, GWP rose by 9–10% from the corresponding baseline results. Exclusion of avoided NH_3_ and NO_3_^−^ increased AP and EP by 4% and 1%, respectively. While these results are sensitive to the soil type and weather and thus cross-site comparisons are not straightforward, the values above are in line with other studies adopting similar approaches (Nguyen et al., 2011, Reckmann et al., 2013).

Finally, changing the assumption regarding on-farm energy usage using the upper and lower limit values reported by McCutcheon (2012) did not greatly affect the baseline results. High energy use resulted in a 2–3% increase in GWP, while the increases in AP and EP were even smaller (1% and <1%, respectively). Low energy usage decreased GWP by 1–2%, with little effect (<1%) observed for AP and EP. These findings suggest that the environmental footprint of pig production systems is not sensitive to the farm's strategy about energy usage.

### 3.6 Comparisons with previous research and system boundaries

A recent study by McAuliffe et al. (2016) suggested that pig LCAs can broadly be categorised into three themes: feed, whole-system, or waste. Of these three themes, Table 7 offers a comparison of the current results with 14 other whole-system studies. Reviewing numerous LCA studies conducted in the area of food production, Roy et al. (2009) posited that cross-study comparisons are difficult due to different model assumptions and system boundaries. Indeed, some studies set the system boundary to the farm gate, while others include the abattoir (Table 7). To navigate this limitation, the current study adopted two functional units (CW and LW), which allowed a broader interpretation of results. For example, Dourmad et al. (2014) report similar values of GWP, AP and EP for France using LW as the functional unit, whereas the GWP and AP values estimated by Nguyen et al. (2011) for Denmark based on CW are also comparable to the present study. Furthermore, while Nguyen et al. (2011) and Halberg et al. (2010) adopt a different metric for EP (g NO_3_-eq), when the baseline EP from the current study is recalculated according to the same impact assessment method (Wenzel et al., 1997), the result (310 g NO_3_/kg CW) is only slightly higher than the value reported by Nguyen et al. (2011) and slightly lower than that by Halberg et al. (2010) (Table 7). It is therefore plausible to conclude that the environmental performance of the Irish pig sector is largely in line with wider European systems. Within the present dataset, the relative performances of the three representative farms were largely unaffected by this change in functional unit, as only small percentages of the overall environmental footprint originate from the slaughtering process (Supplementary Tables S3–S5). Finally, it is worthwhile noting that a recent worldwide analysis of pig supply chains (MacLeod et al., 2013) predicted that GWP values for Western European systems were in a region above 6 kg CO_2_-eq/kg CW, higher than many of the studies presented in Table 7. However, this discrepancy is largely attributable to the fact that MacLeod et al. (2013) fully (and thus perhaps excessively) account for LUC from soybean cultivation, rather than different functional units or system boundaries.

**Table 7:** Comparisons of the present results with previous pig LCA studies.

Study	Scope	Functional unit	GWP	AP	EP
Basset-Mens and Van der Werf (2005)	Crop production to pig farm gate	1 kg liveweight	2.3 kg CO_2_-eq	43.5 g SO_2_-eq	20.8 g PO_4_-eq
Williams et al. (2006)	Crop production to pig farm gate	1000 kg carcass weight	6400 kg CO_2_-eq	394 kg SO_2_-eq	100 kg PO_4_-eq
Dalgaard et al. (2007)	Crop production to delivery of pork to Port Harwich in Britain	1 kg pork	3.6 kg CO_2_-eq	45 g SO_2_-eq	232 g NO_3_-eq
Perez (2009)	Crop production to pig farm gate	1000 kg liveweight	3284.3 kg CO_2_-eq	43.8 kg SO_2_-eq	192.6 NO_3_-eq
Wiedemann et al. (2010)	Crop production to slaughterhouse	1 kg carcass weight at the meat processor gate	5.5 kg CO_2_-eq	N/A	N/A
Halberg et al. (2010)	Crop production to pig farm gate	1 kg liveweight	3320 g CO_2_-eq	61.4 g SO_2_-eq	381 g NO_3_-eq
Nguyen et al. (2010)	Crop production to pig farm gate	1 kg slaughter weight	4812 g CO_2_-eq	N/A	N/A
Pelletier et al. (2010)	Crop production to pig farm gate	1 kg liveweight	2.5 kg CO_2_-eq	N/A	15.9 g PO_4_-eq
Nguyen et al. (2011)	Crop production to slaughterhouse gate	1 kg pork delivered from the slaughterhouse	3.1 kg CO_2_-eq	56 g SO_2_-eq	243 g NO_3_-eq
Devers et al. (2012)	Crop production to delivery of pork to Antwerp in Belgium	1 kg carcass weight	2.6 kg CO_2_-eq	39 g SO_2_-eq	22 g PO_4_-eq
Dolman et al. (2012)	Crop production to pig farm gate	100 kg liveweight	546 kg CO_2_-eq	5.3 kg SO_2_-eq	61.4 kg NO_3_-eq
Jacobsen et al. (2013)	Crop production to meat processor gate	1 kg deboned pigmeat	4.8 kg CO_2_-eq	N/A	N/A
Reckmann et al. (2013)	Crop production to slaughterhouse gate	1 kg pork slaughter weight	3.2 kg CO_2_-eq	57.1 g SO_2_-eq	23.3 PO_4_-eq
Dourmad et al. (2014)	Crop production to pig farm gate	1 kg liveweight	2.3 kg CO_2_-eq	44 g SO_2_-eq	18.5 PO_4_-eq
Current study	Crop production to pig farm gate	1 kg liveweight	2.4 kg CO2-eq	32.6 g SO2-eq	21.4 g PO4-eq
Current study	Crop production to slaughterhouse gate	1 kg carcass weight	3.5 kg CO2-eq	43.8 g SO2-eq	32.1 g PO4-eq

### 3.7 The global context

It is estimated that as much as 36% of energy produced by the world's crops are being used for animal feed, of which only 12% subsequently enter the human diet (Cassidy et al., 2013). Discussing necessary steps to realise global food security, Eisler et al. (2014) asserted the need to replace human-edible crops currently consumed by ruminants with human-inedible feeds such as grasses and pasture legumes. This challenge has an immediate and direct consequence on monogastric livestock systems around the world, which cannot necessarily adopt the same strategy to improve their production efficiency.

Previous research has shown that environmentally focused inclusion of SAA to feed formula can further reduce CP requirements in pigs through a targeted delivery of essential amino acids to counteract basal diet deficiencies (Osada et al., 2011). This reduction in CP is associated with lower GWP, AP and EP at both the feed production stage and during manure management, and likely creates further opportunities for improved environmental efficiencies (Garcia-Launay et al., 2014, Mosnier et al., 2011, Ogino et al., 2012). Regarding waste management, seemingly the most promising technology for reducing environmental impacts is anaerobic digestion of manures (McAuliffe et al., 2016). However, in order to make the system feasible at the global scale, issues such as the shortage of digestion plants and unappealing tariffs for selling energy back to the public grid must first be addressed (Nolan et al., 2012).

## 4 Conclusion

In this study, the LCA method was applied to commercial pig production in RoI and, in addition to the baseline analysis, a range of scenario and sensitivity analyses were conducted. For the average representative farm, GWP, AP and EP were estimated to be 3.5 kg CO_2_-eq kg CW^−1^, 43.8 g SO_2_-eq kg CW^−1^ and 32.1 g PO_4_-eq kg CW^−1^, respectively. Economically efficient herds demonstrated environmental improvements of up to 6% for GWP, 12% for AP and 15% for EP. Feed produced by a small-scale on-farm mill resulted in a lower GWP primarily due to more extensive usage of wheat and barley (rather than maize bran which required further processing), while AP and EP were elevated as a result of higher CP contents. The trade-offs demonstrated by the present study, namely those between high-energy and high-protein diets, have a globally important policy bearing that local environmental conditions, for example the existing level of water quality and catchment-level topography, must be considered when assessing recommendations on optimal production strategies. In other words, globally comparable results of LCA outputs should not be interpreted as a sign that optimal mitigation strategies are also globally comparable.

The results presented here suggest that improvements in on-farm production efficiency will generally also improve environmental sustainability of pig production. The efficiency-environment link identified here is likely to be also applicable to the majority of indoor operations around the world. However, further research is required to recognise the exact nature of this correlation, and particularly when and how certain feeding strategies and waste management technologies, such as the options discussed above, should be employed. In all likelihood, a combination of improvements in feed formulation, farm operation and off-farm waste management will be the key to ensuring sustainable pig production.
