# Supplementary material for: Environmental trade-offs of pig production systems under varied operational efficiencies
Source: J Clean Prod. 2017 Nov 1;165:1163–73. doi: 10.1016/j.jclepro.2017.07.191 (PMC5589118; doi:10.1016/j.jclepro.2017.07.191)
Supplement: Supplementary file 1 [file mmc1.pdf]

Environmental trade-offs of pig production systems under varied operational efficiencies

G. A. McAuliffe[1,2], T. Takahashi[1,2*], L. Mogensen[3], J. E. Hermansen[3], C. L. Sage[4], D. V. Chapman[5] and M. R. F. Lee[1,2]

[1] Rothamsted Research, North Wyke, Okehampton, Devon EX20 2SB, UK
[2] School of Veterinary Sciences, University of Bristol, Langford, Somerset BS40 5DU, UK
[3] Department of Agroecology, Aarhus University, Blichers Allé 20, DK-8830 Tjele, Denmark
[4] Department of Geography, University College Cork, Donovan's Road, Cork, Ireland
[5] School of Biological, Earth and Environmental Sciences, University College Cork, Distillery Field, North Mall, Cork, Ireland

*Corresponding author. Tel: +44 1837 883558. Email: taro.takahashi@rothamsted.ac.uk

Supplementary material (11 pages)

List of abbreviations:

AVG: average herd performance

T25: top 25% herd performance

T10: top 10% herd performance

GWP: global warming potential

AP: acidification potential

EP: eutrophication potential

Table S1. Life cycle inventory for slaughterhouse process.

	Unit	T10	T25	AVG
Inputs				
Liveweight	kg	1000	1000	1000
Electricity	kWh	248.9	248.4	251.6
Diesel	kg	7.4	7.4	7.5
Transport	km	50	50	50
Outputs				0.0
Carcass weight	kg	761.7	763.4	762.4
Losses				
CO	g	2.8	2.8	2.8
CO_2	kg	42.1	42.1	42.6
NO_x	g	27.9	27.8	28.2
N_2O	g	0.7	0.7	0.8
CH_4	g	0.8	0.8	0.8
BOD_5	g	879.5	877.8	888.9
COD	kg	22.9	22.8	23.1
N	kg	3.0	3.0	3.0
P	g	263.7	263.3	266.6
Biodegradable waste	kg	3.7	3.7	3.8

Table S2. Uncertainty parameters adopted in Monte Carlo analysis.

Emission	Uncertainty	Distribution	Reference
CH_4 (enteric fermentation)	± 17%	Triangular	Duffy et al. (2017)
CH_4 (manure management)	± 19%	Triangular	
N_2O (all emissions)	2 (SD^2)	Lognormal	IPCC (2006)
NH_3 (all emissions)	± 21%	Triangular	Amon et al. (2016)
NO_X (all emissions)	2 (SD^2)	Lognormal	
NO_3	1.58 (SD^2)	Lognormal	Pedigree matrix
PO_4	1.58 (SD^2)	Lognormal	(Muller et al., 2016)

Table S3. Environmental impacts for the average (AVG) farm based on the baseline analysis.

Item	GWP (g CO_2-eq)	AP (g SO_2-eq)	EP (g PO_4-eq)
Feed production and transportation			
Dry sow	237	2.3	2
Lactating sow	54.6	0.5	0.4
Gilt	42.4	0.4	0.4
Weaner	388	3.4	2.9
Finisher	1310	12.7	10.5
Transport of feed from mill to farm			
By truck	29.5	0.1	< 0.1
Energy use			
Electricity	130	0.5	0.4
Heat (oil)	51	0.1	< 0.1
Farm emissions			
Methane			
Enteric fermentation	178.5	-	-
Manure management	813.1	-	-
Nitrous oxide	118	-	< 0.1
Ammonia	-	11.3	2.5
Nitrogen oxides	-	0.4	< 0.1
Manure utilisation			
Transport	36	0.3	< 0.1
Spreading	15.9	< 0.1	< 0.1
Nitrous oxide	262	-	
Ammonia	-	12.2	2.7
Nitrogen oxides	-	< 0.1	< 0.1
Nitrate	-	-	5.9
Phosphate	-	-	0.3
Avoided fertiliser production			
from manure nitrogen	-334	-	-
from manure phosphorus	-39.4	-	-
from manure potassium	-27.4	-	-
Avoided fertiliser application			
Spreading	-1.6	- < 0.1	- < 0.1
Nitrous oxide	-62.8	-	- < 0.1
Ammonia	-	-1.7	-0.4
Nitrogen oxides	-	-0.2	- < 0.1
Slaughterhouse			
Electricity	239	1	0.6
Diesel	5.7	< 0.1	< 0.1
Transport	3.9	< 0.1	< 0.1
Landfill	2.5	< 0.1	< 0.1
Emissions (aggregated)	50	0.1	3.4

Table S4. Environmental impacts for the T25 farm based on the baseline analysis.

Item	GWP (g CO_2-eq)	AP (g SO_2-eq)	EP (g PO_4-eq)
Feed production and transportation			
Dry sow	227	2.2	1.9
Lactating sow	51.1	0.5	0.4
Gilt	41	0.4	0.3
Weaner	385	3.5	2.9
Finisher	1160	11.2	9.3
Transport of feed from mill to farm			
By truck	27	0.1	< 0.1
Energy use			
Electricity	128	0.5	0.3
Heat (oil)	50.3	0.1	< 0.1
Farm emissions			
Methane			
Enteric fermentation	171.6	-	-
Manure management	780.6	-	-
Nitrous oxide	100.7	-	< 0.1
Ammonia	-	9.7	2.2
Nitrogen oxides	-	0.3	< 0.1
Manure utilisation			
Transport	30.7	0.2	< 0.1
Spreading	13.6	< 0.1	< 0.1
Nitrous oxide	223.8	-	0.1
Ammonia	-	10.4	2.2
Nitrogen oxides	-	< 0.1	< 0.1
Nitrate	-	-	5.1
Phosphate	-	-	0.2
Avoided fertiliser production			
from manure nitrogen	-285	-	-
from manure phosphorus	-29	-	-
from manure potassium	-24.9	-	-
Avoided fertiliser application			
Spreading	-1.4	- < 0.1	- < 0.1
Nitrous oxide	-53.6	-	- < 0.1
Ammonia	-	-1.5	-0.3
Nitrogen oxides	-	-	- < 0.1
Slaughterhouse			
Electricity	236	1	0.6
Diesel	5.6	< 0.1	< 0.1
Transport	3.8	<0.1	< 0.1
Landfill	2.5	< 0.1	< 0.1
Emissions (aggregated)	60	0.1	3.4

Table S5. Environmental impacts for the T10 farm based on the baseline analysis.

Item	GWP (g CO_2-eq)	AP (g SO_2-eq)	EP (g PO_4-eq)
Feed production and transportation			
Dry sow	216	2.1	1.8
Lactating sow	52.5	0.5	0.4
Gilt	39	0.4	0.3
Weaner	340	3.1	2.6
Finisher	1200	11.6	9.6
Transport of feed from mill to farm			
By truck	26.8	0.1	< 0.1
Energy use			
Electricity	129	0.5	0.3
Heat (oil)	50.6	0.1	< 0.1
Farm emissions			
Methane			
Enteric fermentation	171.3	-	-
Manure management	789.6	-	-
Nitrous oxide	97.4	-	< 0.1
Ammonia	-	9.4	2
Nitrogen oxides	-	0.3	< 0.1
Manure utilisation			
Transport	29.7	0.2	< 0.1
Spreading	13.2	< 0.1	< 0.1
Nitrous oxide	215.8	-	0.1
Ammonia	-	10.1	2.2
Nitrogen oxides	-	< 0.1	< 0.1
Nitrate	-	-	4.9
Phosphate	-	-	0.2
Avoided fertiliser production			
from manure nitrogen	-276	-	-
from manure phosphorus	-27.7	-	-
from manure potassium	-24.5	-	-
Avoided fertiliser application			
Spreading	-1.3	- < 0.1	- < 0.1
Nitrous oxide	-51.8	-	- < 0.1
Ammonia	-	-1.4	-0.3
Nitrogen oxides	-	-0.1	- < 0.1
Slaughterhouse			
Electricity	237	1	0.6
Diesel	5.6	< 0.1	< 0.1
Transport	3.9	< 0.1	< 0.1
Landfill	2.5	< 0.1	< 0.1
Emissions (aggregated)	60	< 0.1	3.4

Table S6. Percentage differences in LCA outputs of scenario and sensitivity analyses relative to the baseline results.

	AVG			T25			T10		
	GWP	AP	EP	GWP	AP	EP	GWP	AP	EP
On-farm feed mill	-11.1	13.5	6.5	-10.9	13.9	6.6	-10.9	13.1	5.9
Domestic wheat and barley	-1.1	0.2	-0.6	-0.9	0.0	-0.7	-0.9	0.3	-0.7
Mass allocation	6.3	5.3	6.5	6.1	5.1	6.6	6.1	5.5	6.6
Inclusion of land use change	80.9	-	-	79.7	-	-	77.9	-	-
High on-farm energy usage	3.1	0.9	0.6	3.3	1.0	0.7	3.3	1.0	0.7
Low on-farm energy usage	-2.0	-0.5	-0.3	-1.8	-0.5	-0.7	-2.1	-0.5	-0.7
Exclusion of fertiliser offsetting	13.1	4.3	1.6	11.8	4.1	1.4	11.5	4.2	1.4

Table S7. Environmental impacts for the average (AVG) farm when feeds are sourced from the on-farm mill.

Item	GWP (g CO_2-eq)	AP (g SO_2-eq)	EP (g PO_4-eq)
Feed production and transportation			
Dry sow	180	1.6	1.4
Lactating sow	44.8	0.4	0.3
Gilt	32.2	0.3	0.3
Weaner	341	2.8	2.4
Finisher	1090	9.7	8.3
Transport of feed from mill to farm			
By truck	-	-	-
Energy use			
Electricity	130	0.5	0.4
Heat (oil)	51	0.1	< 0.1
Farm emissions			
Methane			
Enteric fermentation	178.5	-	-
Manure management	813.1	-	-
Nitrous oxide	174.1	-	0.1
Ammonia	-	16.9	3.7
Nitrogen oxides	-	0.6	0.1
Manure utilisation			
Transport	53.1	0.4	< 0.1
Spreading	23.5	0.1	< 0.1
Nitrous oxide	386	-	0.3
Ammonia	-	17.9	4
Nitrogen oxides	-	< 0.1	< 0.1
Nitrate	-	-	8.8
Phosphate	-	-	0.5
Avoided fertiliser production			
from manure nitrogen	-493	-	-
from manure phosphorus	-68.2	-	-
from manure potassium	-37.4	-	-
Avoided fertiliser application			
Farm traction	-2.4	- < 0.1	- < 0.1
Nitrous oxide	-92.8	-	- < 0.1
Ammonia	-	-2.5	-0.5
Nitrogen oxides	-	-0.2	- < 0.1
Slaughterhouse			
Electricity	239	1	0.6
Diesel	5.7	< 0.1	- < 0.1
Transport	3.9	< 0.1	- < 0.1
Landfill	2.5	< 0.1	- < 0.1
Emissions (aggregated)	60	< 0.1	3.4

Table S8. Environmental impacts for the T25 farm when feeds are sourced from the on-farm mill.

Item	GWP (g CO_2-eq)	AP (g SO_2-eq)	EP (g PO_4-eq)
Feed production and transportation			
Dry sow	173	1.6	1.4
Lactating sow	41.9	0.4	0.3
Gilt	31.1	0.3	0.2
Weaner	339	2.8	2.4
Finisher	967	8.6	7.4
Transport of feed from mill to farm			
By truck	-	-	-
Energy use			
Electricity	128	0.5	0.3
Heat (oil)	50.3	0.1	< 0.1
Farm emissions			
Methane			
Enteric fermentation	171.6	-	-
Manure management	780.6	-	-
Nitrous oxide	152.2	-	< 0.1
Ammonia	-	14.7	3.2
Nitrogen oxides	-	0.6	< 0.1
Manure utilisation			
Transport	46.5	0.3	< 0.1
Spreading	20.6	0.1	< 0.1
Nitrous oxide	338	-	0.3
Ammonia	-	15.7	3.5
Nitrogen oxides	-	< 0.1	< 0.1
Nitrate	-	-	7.7
Phosphate	-	-	0.4
Avoided fertiliser production			
from manure nitrogen	-431	-	-
from manure phosphorus	-55.6	-	-
from manure potassium	-34.1	-	-
Avoided fertiliser application			
Farm traction	-2.1	- < 0.1	- < 0.1
Nitrous oxide	-81.1	-	- < 0.1
Ammonia	-	-2.2	-0.5
Nitrogen oxides	-	-0.2	- < 0.1
Slaughterhouse			
Electricity	236	1	0.6
Diesel	5.6	< 0.1	< 0.1
Transport	3.8	< 0.1	< 0.1
Landfill	2.5	< 0.1	< 0.1
Emissions (aggregated)	50	< 0.1	3.3

Table S9. Environmental impacts for the T10 farm when feeds are sourced from the on-farm mill.

Item	GWP (g CO_2-eq)	AP (g SO_2-eq)	EP (g PO_4-eq)
Feed production and transportation			
Dry sow	164	1.5	1.3
Lactating sow	43.1	0.4	0.3
Gilt	29.6	0.3	0.2
Weaner	299	2.4	2.1
Finisher	1000	8.9	7.7
Transport of feed from mill to farm			
By truck	-	-	-
Energy use			
Electricity	129	0.5	0.3
Heat (oil)	50.6	0.1	< 0.1
Farm emissions			
Methane			
Enteric fermentation	171.3	-	-
Manure management	789.6	-	-
Nitrous oxide	146.4	-	< 0.1
Ammonia	-	14.2	3.1
Nitrogen oxides	-	0.5	< 0.1
Manure utilisation			
Transport	44.8	0.3	< 0.1
Spreading	19.8	< 0.1	< 0.1
Nitrous oxide	326	-	0.3
Ammonia	-	15.2	3.3
Nitrogen oxides	-	< 0.1	< 0.1
Nitrate	-	-	7.4
Phosphate	-	-	0.4
Avoided fertiliser production			
from manure nitrogen	-416	-	-
from manure phosphorus	-53.9	-	-
from manure potassium	-33.6	-	-
Avoided fertiliser application			
Farm traction	-2	- < 0.1	- < 0.1
Nitrous oxide	-78.1	-	- < 0.1
Ammonia	-	-2.1	-0.5
Nitrogen oxides	-	-0.2	- < 0.1
Slaughterhouse			
Electricity	237	1	0.6
Diesel	5.6	< 0.1	< 0.1
Transport	3.9	< 0.1	< 0.1
Landfill	2.5	< 0.1	< 0.1
Emissions (aggregated)	50	< 0.1	3.4

References

Amon, B., Hutchings, N., Dämmgen, U. & Webb, J. 2016. EMEP/EEA air pollutant emission inventory Guidebook 2016. Copenhagen: European Environment Agency.

Duffy, P., Black, K., O'Brien, P., Hyde, B., Ryan, A. M., Ponzi, J. & Alam, S. 2017. National Inventory Report 2017: Greenhouse Gas Emissions 1990-2015 Reported to the United Nations Framework Convention on Climate Change. Wexford: Environmental Protection Agency.

IPCC 2006. Guidelines for National Greenhouse Gas Inventories. Hayama, Japan: The Greenhouse Gas Inventories Programme, The Intergovernmental Panel on Climate Change.

Muller, S., Lesage, P., Ciroth, A., Mutel, C., Weidema, B. P. & Samson, R. 2016. The application of the pedigree approach to the distributions foreseen in ecoinvent v3. International Journal of Life Cycle Assessment, 21**,** 1327-1337.
